# Electro-Therapeutics

**Published:** 1879-10-20

**Authors:** William F. Hutchinson

**Affiliations:** Providence, R. I.


### ELECTRO-THERAPEUTICS.

BY WILLIAM F. HUTCHINSON, A.M., M.D., PROVIDENCE, R. I.

Where the galvanic bath is not available, and, on account of the great cost of its establishment and maintainance, it is rarely so, we must fall back upon general galvanization to obtain similar effects at a much greater expenditure of time and trouble. That is to say, what may be done in the bath by one or two weekly applications, requires daily work in this way.

With the sixteen-cell battery already described, a stool with the center of the seat replaced by a felt covered copper plate, and two or three broad carbon points covered with wash leather and a foot tub of hot water, we are ready for this form of sitting.

It is best to have the patient entirely undressed, and then covered with a loose flannel dressing-gown, which will at once protect from exposure and allow free access to all parts of the body. Each patient should bring his or her own dressing-gown, for which a wardrobe may be provided. Moisten thoroughly the felt-covered plate in the stool with a weak solution of salt in water as hot as can be comfortably borne, and seat your patient thereupon in such a way that the gown will cover his legs, feet and the stool. Connect the plate by a set-screw with the negative pole of the battery, attach a broad carbon point, thoroughly wet, to the other cord, and begin with four cells to stroke downward the spinal column from the fifth cervical vertebra to the sacrum. Do not go higher, or flashes, indicating cerebral stimulus, will be produced. Gradually increase the tension until a sharp tingling is felt, and continue, always stroking downward, until the skln is thoroughly reddened, probably about seven or eight minutes.

Although Remak and Benedikt place great importance upon the current direction, I have been unable to satisfy myself that it makes any essential difference in results in general galvanization, and am of the opinion that it is immaterial which pole is uppermost.

Next, change the hand electrode to the epigastrium over the solar plexus, and let it remain for three or four minutes. Then the long thoracic nerve and its tracts of supply on each side, and change the stool connection for one with a plate, which I have set in the side of the foot tub, or to another electrode, which may be simply dropped into the water. This, with the patient holding the first carbon point in either hand until the palm is aglow and perspiring, a period of eight or ten minutes, finishes the sitting.

Too much stress cannot be laid upon two cardinal points in galvanic or electrical treatment, and if these be neglected, not only will the most persistent work be barren of good results, but absolute harm may, and sometimes does, follow. Never use a current strong enough to cause pain, nor make a sitting long enough to tire a patient.

Two years ago, while watching my friend Dr. Onimus, of Paris, at work, I was surprised to see the very short time, rarely exceeding six minutes, alloted to each patient, and still more to hear him say that his results were better than formerly when he made longer applications. Although I did not agree with him altogether, I am satisfied that all the benefit to be obtained from a galvanic application can be gotten in a much less space of time than that usually employed, and that many ill effects which have been attributed to the use of galvanism are due to its unskilful handling, of which one chief evidence is fatigue arising from too long a sitting.

I have found the value of general galvanization most evident in cases of neurasthenia Americana, the variety of nerve exhaustion to which our women seem more liable than their sisters in other lands, and which, from the invariability of its train of symptoms not seen as a whole in other countries, led me to name the disease, or rather absence of normal force, from the place which has had the melancholy honor of being its home. No practitioner is unacquainted with its most annoying and persistent victim.

Anæmic, meagre and sallow, narrow-shouldered and wide-jointed, with lustreless eyes, decayed teeth, brittle hair, and a skin covered with pimples, she is actually suffering from generations of mince pie, saleratus biscuits and pork.

The sins of the father shall be visited upon the children, and these aches, this debility, this absolute cumbering of the ground mammal is the direct outgrowth of a hundred years of systematic neglect of all sanitary law. She generally comes from the country, has neuralgia in every square inch of her body, a yarn to spin about her ailments as long as the sailor's top-gallant brace, and has been the terror of every doctor whose office was within her reach, Her metropolitan sister is merely a modified form of the same genus, changed in externals by dress and surroundings, but the same *au fond;* and they are both uncomfortable cases to handle. So they finally drop into the specialist's hands, and are at least valuable as "striking examples" upon which to exhibit the marvellous vitalizing effects of galvanism.

With a rigid diet and sanitary law, strictly enforced, and a daily sitting of the kind above described, they frequently recover with a rapidity that is simply disgusting to the doctors who have in vain expended upon them hours of time and pounds of drugs.—*Med. and Surgical Brief.*

